# Snhg12 targets miR-199a-5p to regulate osteogenic differentiation of TDSCs via the Fzd4/Wnt/β-catenin pathway

**DOI:** 10.1080/15476286.2025.2518754

**Published:** 2025-06-24

**Authors:** Xinyue Li, Binbin Xu, Zhongjie Wang, Mengyi Li, Hao Wu, Bao Chen

**Affiliations:** a School of Medicine, Shanghai Sixth People’s Hospital Affiliated to Shanghai Jiao Tong University, Shanghai, P. R. China; b Department of Thoracic Surgery, Shanghai Chest Hospital, Shanghai Jiaotong University, Shanghai, P. R. China; c Department of Operating Room, Shanghai General Hospital, Shanghai Jiao Tong University School of Medicine, Shanghai, P. R. China; d The University of Sydney Business School, NSW, Australia; e Department of Orthopaedics, The Second Hospital of Lanzhou University, Lanzhou, P. R. China

**Keywords:** Heterotopic ossification, LncRNA Snhg12, miR-199a-5p, TDSCs, FZD4

## Abstract

Heterotopic ossification (HO) is the formation of bone tissue outside the normal skeletal structure following musculoskeletal injury. Long non-coding RNAs (lncRNAs) play a regulatory role in guiding stem cell differentiation towards osteogenic lineages. Through lncRNA sequencing in this study, we observed an up-regulation of small nucleolar RNA host gene 12 (Snhg12) in HO tissues. The precise function of Snhg12 in the process of tendon stem cells (TDSCs) osteogenic differentiation remains uncertain. Our findings suggest that Snhg12 overexpression exacerbated HO, whereas its suppression ameliorated HO. The lncRNA Snhg12 directly targeted miR-199a-5p to alleviate the suppression of Fzd4 caused by miR-199a-5p. Functionally, experiments conducted in vitro and in vivo demonstrated that HO formation was inhibited by the down-regulation of Fzd4 through the up-regulation of miR-199a-5p. In rescue experiments conducted in vitro, the inhibition of miR-199a-5p or overexpression of Fzd4 reversed the improvement in HO caused by Snhg12 knockdown, and Fzd4 regulated the process of osteogenic differentiation in TDSCs by the Wnt/β-catenin pathway. Taken together, these results demonstrate that Snhg12 modulates HO formation via the Snhg12-miR-199a-5p-Fzd4/Wnt/β-catenin signalling pathway.

## Introduction

Abnormal bone formation in soft tissues, like ligaments, muscles, and tendons, known as HO, frequently occurs after skeletal muscle trauma, severe burns, or hip replacement surgery [1]. Frequent occurrence of HO causes long-lasting discomfort and reduces ability to move joints, which causing significant decline in patients’ quality of life [2]. Studies have reported that 65–91% of HO cases arise post-trauma as a severe complication [3,4], 1.5–8% secondary to burns [5] and 13.6% occured after severe brain injury [6]. Although non-steroidal anti-inflammatory medications and local radiation have been employed to prevent and treat HO, clinical date indicate their efficacy is restricted, and necessitating surgery as the primary approach for [7–9]. Despite surgical excision being able to remove heterotopic ossification tissue, post-operative recurrence rates remain high. Hence, understanding the underlying mechanisms of HO to discover effective therapeutic programmes is necessary.

TDSCs are extracted from tendon tissues and possess inherent stem cell properties such as clonogenicity, pluripotency, and the ability to self-renew [10,11]. When tendon tissue is damaged, TDSCs are stimulated by local inflammation and other related factors to differentiate and repair the damaged tissue [12]. The paradigm of stem cell differentiation was previously thought to be endlessly unidirectional and stratified, but its fate is now found to be unstable, subject to biochemical and biomechanical stimuli including those that alter the direction of differentiation [13]. In some studies, it has been suggested that some TDSCs may differentiate towards osteogenesis in response to tendon injury and stimulation of the local tissue microenvironment and have a supportive function in the formation of HO [14,15]. However, there are limited studies in vivo on the involvement of TDSCs in HO and the mechanisms participating in the regulation of HO. Hence, our research concentrates on exploring the role of TDSCs in HO development and elucidating the underlying mechanisms in hopes of expediting the discovery of a cure for HO.

Long non-coding RNA (lncRNA) refers to an RNA molecule exceeding 200 nucleotides in length [16]. Despite lacking protein-coding capacity, lncRNAs regulate the expression levels of other genes [17]. Recent research has uncovered that as much as 90% of the human genome consists of non-coding proteins that also play crucial roles, and lncRNAs play a role in regulating gene expression during transcription, as well as in mRNA editing and processing after transcription [18]. Studies have shown that multiple lncRNAs facilitate the differentiation of stem cells into osteogenic lineages. For instance, lncRNA DANCR enhances the osteogenic differentiation of bone marrow mesenchymal stem cells (BMSCs) by activating the miR-320a/wnt/β-catenin pathway, while lncRNA H19 activates the miR-532-3p/SIRT1 pathway to promote osteogenesis [19,20]. Nevertheless, no action has been taken regarding the involvement of lncRNAs in the osteogenic differentiation of TDSCs or their contributions to HO.

Snhg12, a long non-coding RNA, is situated in the chromosome 1 region of 1p35.3. Previous reports have mentioned the presence of Snhg12 in diseases related to tumours [21], and its involvement in the process of osteogenesis [22]. Nevertheless, the investigation of its impact on the osteogenic differentiation of TDSCs and the development of HO has not yet been explored. Following the high-throughput sequencing of HO tissues, we observed an elevation of Snhg12 in the progression of HO when compared to the sham operation group. Therefore, we targeted Snhg12 to investigate its intrinsic mechanism in the development of HO.

Through high-throughput sequencing and subsequent qPCR assays, we discovered elevated of Snhg12 expression in HO tissues. Furthermore, increasing Snhg12 expression in vivo promoted the formation of HO. The aim of this study is to reveal the potential regulatory mechanisms of Snhg12 in TDSCs. Experiments discovered that increasing Snhg12 promoted osteogenic differentiation of TDSCs. Furthermore, computational predictions by StarBase unveiled Snhg12’s targeting of miR-199a-5p, subsequently modulating the Fzd4/β-catenin pathway. Collectively, our findings shed light on the involvement of Snhg12 in HO development and its potential regulatory mechanisms, enhancing our understanding of the roles of long non-coding RNAs in the context of HO.

## Material and methods

### Regent

Foetal bovine serum (FBS, Cat# 10091148) and Opti-MEMTM (Cat# 31985,070) was supplied by Gibco (CA, USA). Penicillin/streptomycin was supplied by HyClone (UT, USA). All antibodies were diluted in antibody dilution Buffer (Cat# A1800, Solarbio) at the following concentrations: anti-Runx2 (Cat# 20700, Chicago, USA), 1:1000; anti-Opn (Cat# 22951, Chicago, USA), 1:1000; anti-β-catenin (Cat# 51067, Chicago, USA), 1:1000; anti-AGO2 (Cat# 32381, Abcam Biotechnology, MA, USA), 1:1000; anti-GAPDH (Cat# GB11002, Servicebio, Wuhan, China), 1:2000; anti-HSP90 (Cat# Ab2928, Abcam Biotechnology, MA, USA), anti-Mouse (Cat# 7076S, Cell Signaling Technology) 1:5000, anti-Rabbit (Cat# 7074S, Cell Signaling Technology) 1:5000.

### Model establishment

According to a prior study conducted by Peterson and colleagues [23], 8–10-week C57BL/6J male mice were purchased from GemPharmatech Company (Nanjing, China), and selected to establish a trauma-induced HO model using burns/incisions. After anaesthetization with 1% pentobarbital sodium, the leg hair of the mice was shaved to fully expose the skin. A 0.5 cm incision was created on the medial side below the posterior ankle, and the Achilles tendon was cleanly cut in the middle without stitching. Finally, Vicchio sutures were used to close the skin incision. A block of aluminium measuring 2 cm × 2 cm × 3 cm and weighing 35 g was heated in water at a temperature of 60°C. It was then placed on the exposed, porous backs of mice for 17 s. For the sham group, only Achilles tendon tissue was exposed without tendon severance and back burn treatment. After euthanizing the mice 10 weeks post-injury, the skin was incised, and soft tissue from the tendon-muscle junction to the root-bone junction was extracted for subsequent processing. Fresh tissues were fixed in 4% formalin. For the HO group, demineralization was necessary, so the fixed tissues were placed in 19% ethylenediaminetetraacetic acid (EDTA) solution for decalcification; the decalcified tissues were further dehydrated and embedded in paraffin. Paraffin sections were longitudinally cut into 5 micrometres and mounted on Thermo Superfrost®Plus slides. Immunofluorescence, H&E staining, and micro-CT were employed to assess the morphological and compositional changes in the tissues. Including C57BL/6J male mice to extract TDSCs, a total of 120 mice were used in this study. Eighty 8–10 weeks old male C57BL/6J mice were completely randomized into 8 groups, each with 10 mice, using a random number table: Snhg12 overexpression control group (vector); Snhg12 overexpression group (OV-Snhg12), Snhg12 knockdown control group (sh-NC); Snhg12 knockdown group (sh-Snhg12); Snhg12 overexpression + agomiR-199a-5p group (OV-Snhg12 + agomiR-199a-5p), Snhg12 overexpression + Fzd4 knockdown group (OV-Snhg12 + sh-Fzd4), Snhg12 knockdown + antagomiR-199a-5p (sh-Snhg12 + antagomiR-199a-5p); Snhg12 knockdown + Fzd4 overexpression group (sh-Snhg12 + OV-Fzd4). Neither the mice nor the researchers were aware of the group assignments during sample with ad libitum access to water and food. All animal experiments and procedures were approved by the Institutional Animal Care and Use Committee (IACUC) of the Sixth People’s Hospital of Shanghai Jiao Tong University. All animal experiments were conducted in accordance with the ARRIVE guidelines. All experimental protocols were carried out in accordance with relevant guidelines and regulations. Collection and data analysis. All mice were housed in a ventilated, dry environment. The sequence of sh-RNA was listed in Table S1.

### LncRNA sequencing

Total RNA was extracted from tissues of both HO model mice and sham-operated control group mice. For acquiring RNA expression profiles, the VAHTS Total RNA-seq (H/M/R) Library Preparation Kit from Vazyme (China) was employed to construct total RNA-seq libraries. Subsequent sequencing of these libraries was performed utilizing the Illumina HiSeq™ 2000 platform provided by Illumina (USA). Data analysis was conducted using the HISAT alignment software, whereby lncRNAs exhibiting a P-value of less than 0.05 were classified as differentially expressed.

### Luciferase assay

Luciferase receptor plasmids were generated by cloning wild-type (WT) Snhg12, mutant-type (MUT) Snhg12, WT-3′-UTR-Fzd4, and MUT-3′-UTR-Fzd4 using PmiRGLO vectors from GenePharma. When TDSCs grew to a cell density of 70% in 24-well plates, lipofectamine 3000 (Invitrogen, USA) was used to co-transfect agomiR-199a-5p, antagomiR-199a-5p, and luciferase receptor plasmids into the TDSCs. After cell culture and transfection, harvested cells were assayed for luciferase activity using the Dual-Luciferase Reporter Assay System (Promega) according to the manufacturer’s instructions.

### RNA immunoprecipitation (RIP) assay

The Magna RIP™ RNA Binding Protein Immunoprecipitation Kit from Millipore (USA) was utilized. The cells were lysed with RIP lysis buffer when the cell density reached 90%. Subsequently, they were incubated with anti-AGO2 antibody in lysis buffer along with magnetic beads. This facilitated the binding of the antibody to the magnetic beads, which were later separated using a magnetic separator. Protein digestion was achieved by introducing proteinase K. As negative controls, plain rabbit IgG from the RIP kit was used. Immunoprecipitated RNA was extracted and assayed by qPCR to confirm enrichment of bind targets.

### Cell culture and osteogenic induction

TDSCs were extracted from tendon tissues of 8 weeks old C57BL/6J male mice. The tissue blocks were then placed in a medium containing 0.05% trypsin (Thermo, USA) at 37°C for about 30 min to digest. After digestion was completed, an equal volume of α-MEM (Gibco, USA) medium was added and centrifuged to collect cell sediment, which was rinsed two times with PBS that had 1% ammonium lactate and purified water. Afterwards, it was suspended in petri dishes measuring 25 cm^^2^ and containing 10% FBS (Gibco, USA) and α-MEM medium. The suspension was then placed in an incubator at 37°C with 5% CO_2_ for 3–4 days. Digest them when a density of cell reach 80–90% fusion. Inoculate TDSCs in six-well plates with about 3x10^3^ cells per well and add 2 ml/well α-MEM. Put into 37°C, 5% CO2 incubator for incubation. Twenty-four hour later, remove the old α-MEM and add 2 ml/well osteogenic induction medium (Gibco, USA). Change the medium every 3 days and induce for 2–3 weeks. After 2–3 weeks, calcium nodules were formed, and alizarin red staining was performed.

### Cell transfection and viral infection

GenePharma (Jiangsu, China) provided the sh-Snhg12, OV-Snhg12, and OV-Fzd4 plasmids, which were then cotransfected into TDSCs using lipofectamine 3000 (Invitrogen, USA). Additionally, GenePharma (Jiangsu, China) supplied agomiR-199a-5p, antagomiR-199a-5p, and their respective negative controls. agomiR-199a-5p and antagomiR-199a-5p were injected into the injury site of C57BL/6J mice at a dose of 20 nmol every fortnight for 10 weeks. Obio Technology (Shanghai, China) provided knockdown and overexpression AAV viruses for Snhg12 and Fzd4. C57BL/6J mice received injections of AAV (3.5 × 10^10 vg per injection) diluted in phosphate buffer solution (PBS) at the injury site every 2 weeks for a total of 10 weeks, with a volume of 25 μl per injection.

### Western blots

Proteins were extracted from tissues and cells using RIPA buffer (Beyotime, China) with 50 mm Tris-HCl at a pH of 8. The BCA kit (Beyotime, China) identified the levels of protein concentrations. To prepare the protein samples, 5× loading Buffer (Beyotime, China) was added, and the mixture was heated at 100°C for 15 min. Protein samples were separated using SDS-PAGE polyacrylamide gel electrophoresis and then transferred onto 0.45um NC membranes. The NC membrane was blocked with 5% BSA at room temperature for 1 h. The membrane was cropped and put in primary antibody (diluted in 5% BSA) and shaken gently at 4°C overnight. The main antibody was retrieved and then mixed with sodium azide (a substance that can hinder bacterial growth) at a concentration of 5ul/ml in the primary antibody solution. The mixture was subsequently stored at 4°C. TBST was used to wash the membrane three times, with each wash lasting 5 min. Add the secondary antibody (generally 1:2000 dilution), room temperature gentle shaking 1 h. The membrane was washed by TBST three times, each time 5 min.

### Real-time qPCR

TRIzol (Thermo Fisher Scientific, USA) was utilized to extract total RNA from cells or tissues. The concentration of RNA was quantified using a NanoDrop spectrophotometer (Thermo Fisher Scientific, USA). Total RNA was transcribed into cDNA using a TaKaRa kit (TaKaRa, China) according to the manufacturer’s instructions. Fast SYBR Green Master Mix (Biosystems, USA) was used for real-time qPCR on a Light Cycler 480 (Roche, Switzerland), all procedures were in accordance with the kit instructions. The calculation of gene expression levels was done using 2-ΔΔCt. The primers were listed in Table S2.

### Alkaline phosphatase staining and activity

TDSCs were spread on 24-well plates and rinsed with preheated α-MEM for 2–3 min 7 days after induction of osteogenesis. The cells were treated with paraformaldehyde for 15 min and subsequently washed two times with PBS for 5-min each. To observe, the cells were identified by employing the Alkaline phosphatase assay kit (cat# A059–2–1 Nanjing jiancheng Bioengineering Institute, China), following the manufacturer’s instructions for the procedure. After adding the chromogenic agent, mix the solution and measure the absorbance at 520 nm wavelength using a SpectraMax M3 microplate reader (Molecular Devices, USA). Utilize the Pierce Protein Assay Kit (Pierce, American) to test the total protein content of the same sample.

### Alizarin red staining

TDSCs were spread on 24-well plates and rinsed with pre-warmed α-MEM for 2–3 min 7 days after induction of osteogenesis. The cells were treated with paraformaldehyde for 15 min, then rinsed twice with PBS for 5 min each, and subsequently subjected to staining with 2% ARS (Sigma-Aldrich, USA) for 30 min at ambient temperature. The cells were dyed for half an hour at ambient temperature. The staining was dissolved using 100 mm hexadecylpyridinium chloride (Sigma-Aldrich) for 1 hour. The absorbance was measured at 562 nm using a SpectraMax M3 microplate reader (Molecular Devices, USA).

### H&E staining

H&E staining was performed using the H&E staining kit (G1120, Solarbio). Sections of deparaffinised skeletal muscle tissue were stained using Mayer’s haematoxylin, followed by eosin staining. After staining with eosin for 50 s, the sections were dehydrated with ethanol (95%, 100%), then cleared with xylene and sealed. Images (Leica, Germany) were obtained by the microscope.

## Immunofluorescence analysis

Cells were cultured in 6-well plates and treated in different ways as indicated. When the cells density reached to 90%, the medium was eliminated, and the cells were washed two times with PBS for 5 min each. Afterwards, the cells were treated with paraformaldehyde for 15 min, and subsequently washed three times with PBS for 5 min per. In order to prevent non-specific binding, the cells were treated with a solution consisting of 1% BSA and 22.52 mg/mL glycine (Gibco, USA) in PBST (PBS +0.1% Tween 20) for 30 min, then permeabilized with 0.25% Triton X-100 for 10 min at ambient temperature. Subsequently, the cells were incubated with a diluted primary antibody (anti-Runx2, 1:200; anti-Opn, 1:100; anti-Fzd4, 1:200) overnight at 4°C. Subsequently, the cells underwent staining with Alexa Fluor 488-conjugated secondary antibodies in a light-free environment. 4, 6-diamino-2-phenylindole (DAPI) was used to stain the nuclei. Rhodamine-conjugated phalloidin from Yeasen Biotechnology (Shanghai, China) was used to stain the cytoskeleton, and the imaging of the cells was obtained by a digital section scanner (Pannoramic MIDI; 3DHISTECH Ltd).

## Micro‐CT scanning

After 10 weeks of Achilles tendon transection and scalding of the posterior back in C57BL/6J mice, extract the hind leg tissues and immerse in a 10% formalin solution for 48 h. The specimens were subsequently analysed and subjected to scanning using the Skyscan 117, a micro-CT scanner known for its high-resolution capabilities, settings parameter to 18 mm isometric resolution and 70 kV. CTvox software was used to acquire and reconstruct three-dimensional images. CTan software (version 1.15.4.0+, Bruker) was utilized to calculate the volume of bone. The dense mass in soft tissue with Hounsfield units exceeding 272 is considered heterotopic bone.

## Statistical analysis

The data is presented as mean ± SEM. Data were collected in Excel 2020 (Microsoft Corporation). GraphPad Prism 9.0 software (GraphPad Software Inc, USA) was utilized to conduct statistical analyses. Group comparisons were conducted through one-way ANOVA, followed by Tukey’s post hoc test for normally distributed data and Kruskal–Wallis tests for non-normally distributed data. The chi-square test was used to analyse categorical data. A p-value less than 0.05 was to be statistically significant.

## Results

### LncRNA Snhg12 expression is up-regulated in trauma-induced tendon stem cells

**Figure 1.**
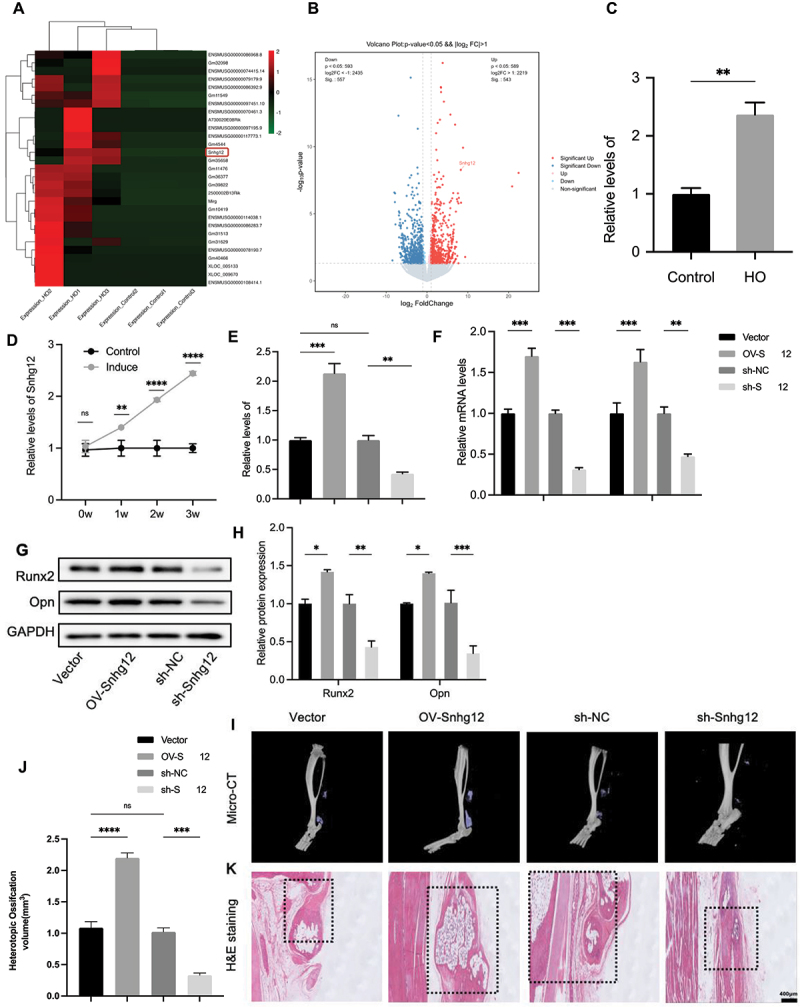
Snhg12 facilitate osteogenic differentiation. (A) Heat maps and (B) The volcano map of differential expressed lncRNAs in tendon tissues from the trauma-induced HO and the sham operation group, red means up-regulation, green/blue mean down-regulation (*n* = 3/group). (C) Relative mRNA levels of Snhg12 in tendon tissues from the trauma-induced HO and the sham operation group were analysed by qPCR (*n* = 3/group). (D) qPCR was used to measure the relative expression levels of Snhg12 in normal and osteogenic induced TDSCs during 3 weeks (*n* = 3/group). (E-K) Overexpress or knockdown Snhg12 in trauma-induced mice using AAV infection for 10 weeks (*n* = 10/group). (E) Relative mRNA levels of Snhg12 in HO tissues were analysed by qPCR (three samples from each group). (F) Relative mRNA levels of Runx2 and Opn were analysed by qPCR (*n* = 3 samples from each group). (G, H) Runx2 and Opn protein levels were analysed by western blots (three samples from each group). (I, J) Mirco-CT and (K) H&E Staining were used to detect osteogenic content of tendon lesions, the regions of the tendon: distal segment (vector), proximal segment (OV-Snhg12), proximal segment (sh-NC), and proximal segment (sh-Snhg12), scale bar: 200 μm (three samples from each group). *p < 0.05; **p < 0.01; ***p < 0.001; ****p < 0.0001.

To elucidate the underlying mechanism heterotopic ossification, high-throughput sequencing was conducted on tendon tissues obtained from both the HO and sham-operated cohorts. The sequencing data revealed a substantial elevation in Snhg12 expression within the HO group (Figure 1A,B). Subsequent assessment of Snhg12 expression levels in the HO and sham-operated groups using the same tissues as in the high-throughput sequencing analysis demonstrated a marked upregulation of Snhg12, as evidenced by qPCR results (Figure 1C).

### Snhg12 facilitate osteogenic differentiation in trauma-induced tendon tissues

Compared to standard growth medium, TDSCs cultured in osteogenic induction medium exhibited a progressively increasing temporal expression pattern of Snhg12 from weeks 0 to 3 (Figure 1D). To further probe the influence of Snhg12 on osteogenic differentiation pathways, we employed Adeno-associated virus serotype 9 (AAV9) as a vector to either overexpress or knockdown Snhg12 (Figure 1E). qPCR (Figure 1F) and western blots (Figure 1G,H) showed a upregulation in the expression of osteogenic biomarkers Runx2 and Opn following Snhg12 overexpression. Additionally, Mirco-CT (Figure 1I,J) and H&E Staining (Figure 1K) showed increased osteogenesis at traumatic tendon sites treated with OV-Snhg12. Conversely, decreasing the expression of Snhg12 yielded the opposite result (Figure 1F–K).

### Snhg12 has a targeting relationship with miR-199a-5p

**Figure 2.**
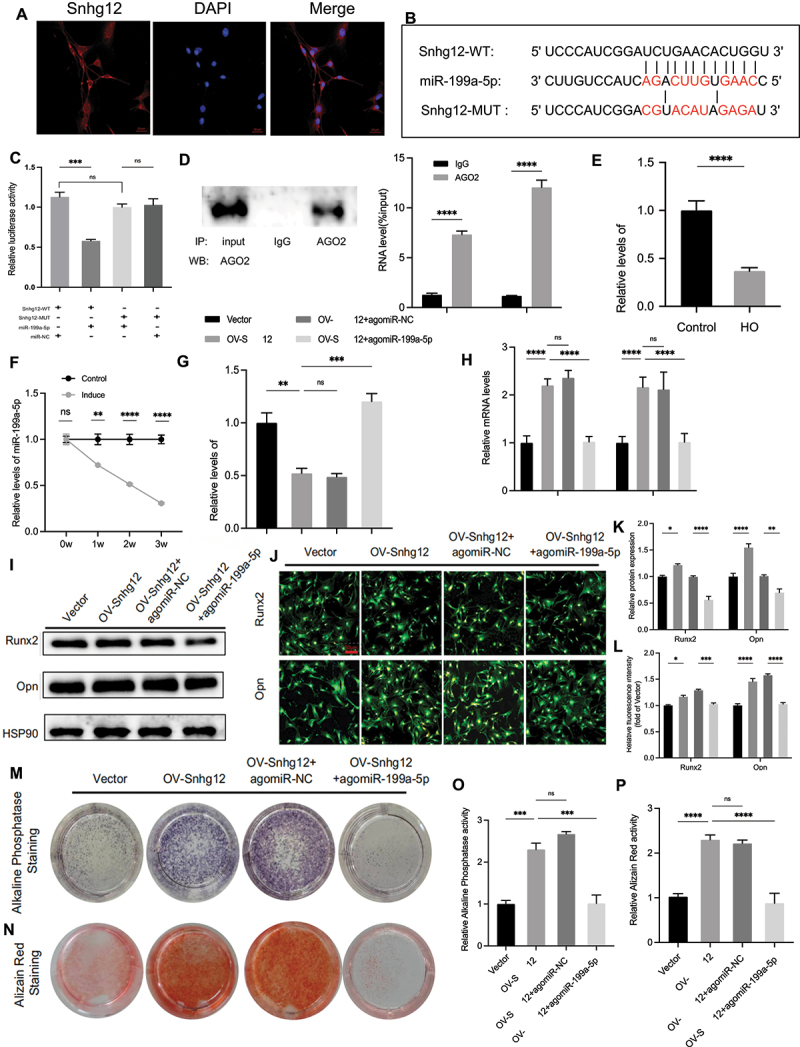
Snhg12 enhances osteogenic differentiation of TDSCs by interacting with miR-199a-5p. (A) The expression site of lncRNA Snhg12 in TDSCs from 8 weeks old C57BL/6J male mice was determined by FISH assay (*n* = 3/group). (B) StarBase software predicted the targeting the sequence of Snhg12. (C) TDSCs were transfected with Snhg12-wt or Snhg12-mut and co-treated with agomiR-NC or agomiR-199a-5p. Relative luciferase was detected using dual luciferase reporter genes (*n* = 3/group). (D) RNA immunoprecipitation assay with anti-AGO2 determined the binding effect of Snhg12 and miR-199a-5p (*n* = 3/group). (E) Relative mRNA expression of miR-199a-5p was detected by qPCR in tendon tissues from the trauma-induced HO and the sham operation group (*n* = 3/group). (F) qPCR was used to measure the relative expression levels of miR-199a-5p in normal and osteogenic induced TDSCs during 3 weeks (*n* = 3/group). (G-P) TDSCs were treated, respectively, with vector, OV-Snhg12, OV-Snhg12+agomiR-NC, OV-Snhg12+agomiR-199a-5p for 2 days. (G) Relative mRNA level of miR-199a-5p was analysed by qPCR (*n* = 3/group). (H) Relative mRNA levels of Runx2 and Opn were analysed by qPCR (*n* = 3/group). (I, K) Runx2 and Opn protein levels were analysed by western blots (*n* = 3/group). (J, L) Immunofluorescence analysis of the expression levels of Runx2 and Opn after transfection and 3 days of osteogenic induction, scale bar: 50 μm (*n* = 3/group). After transfecting the plasmid and osteogenic induction 7–14 days later, (M) alkaline phosphatase and (N) alizarin red staining was performed. (O) Alkaline phosphatase and (P) alizarin red quantification (*n* = 3/group). *p < 0.05; **p < 0.01; ***p < 0.001; ****p < 0.0001.

Given the pivotal role of miRNA-mediated ceRNA interactions, potentially by regulated lncRNA, we utilized fluorescent in situ hybridization to illustrate the predominant cytoplasmic localization of Snhg12 in C57BL/6J tendon-derived stem cells (Figure 2A). Furthermore, leveraging the StarBase software, we predicted potential binding targets for Snhg12, unveiling the targeting relationship between miR-199a-5p and the Snhg12 sequence (Figure 2B). Furthermore, Snhg12 promotes tumourigenesis and metastasis in hepatocellular carcinoma [24], and vascular smooth muscle cell proliferation and migration via targeting miR-199a-5p [25]. In order to validate the binding association between miR-199a-5p and Snhg12 in TDSCs, we performed a luciferase reporter gene assay, which demonstrated that treatment with agomiR-199a-5p decreased the luciferase activity induced by Snhg12-WT, while it had no impact on the luciferase activity induced by Snhg12-MUT (Figure 2C). AGO2, a critical miRNA effector protein, plays a pivotal role in gene silencing through RISC formation. Therefore, we conducted a RIP experiment and found that AGO2 bound to both miR-199a-5p and Snhg12 (Figure 2D).

### The interaction between Snhg12 and miR-199a-5p promotes the osteogenic differentiation of TDSCs

qPCR analysis revealed a significant reduction in miR-199a-5p levels in trauma-induced tendon tissues compared to sham-operated tendon tissues (Figure 2E). Compared to standard growth medium, TDSCs cultured in osteogenic induction medium exhibited a progressively decreasing temporal expression pattern of miR-199a-5*p* from weeks 0 to 3 (Figure 2F). By manipulating miR-199a-5p expression in osteogenically induced TDSCs using agomiR-199a-5p, and influencing Snhg12 expression with OV-Snhg12, it was found that the treatment with OV-Snhg12 led to a reduction of miR-199a-5p, compared to vector group, but the reduction was subsequently counteracted by agomiR-199a-5p (Figure 2G). qPCR (Figure 2H), western blots (Figure 2I,K), and immunofluorescence (Figure 2J,L) results showed up-regulation of biological marker Runx2 and Opn expression in osteogenic differentiation of TDSCs treated with OV-Snhg12, but agomiR-199a-5p reversed the up-regulation. Alkaline phosphatase (Figure 2M,O) and alizarin red staining had similar results (Figure 2N,P). Therefore, Snhg12 facilitates osteogenic differentiation in TDSCs through modulating of miR-199a-5p expression.

### miR-199a-5p regulates osteogenic differentiation of TDSCs through FZD4/β-catenin pathway

**Figure 3.**
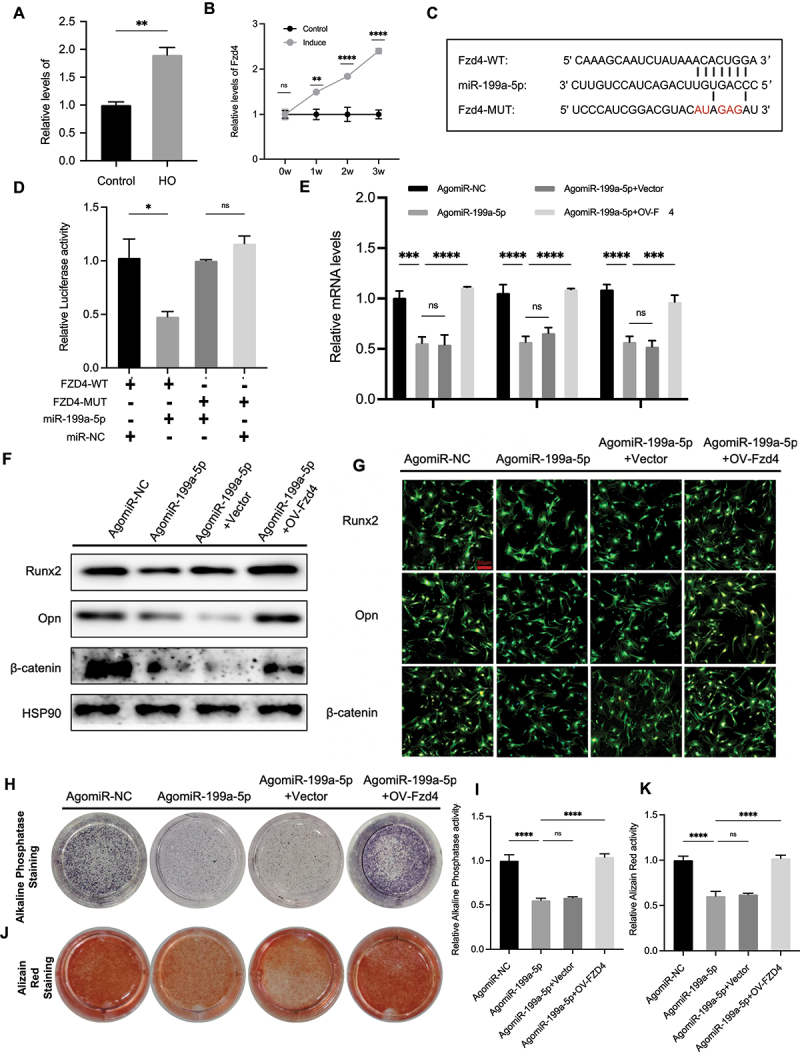
miR-199a-5p regulates osteogenic differentiation of TDSCs through Fzd4/β-catenin pathway. (A) Relative mRNA levels of Fzd4 in tendon tissues from the trauma-induced HO and the sham operation group were analysed by qPCR (*n* = 3/group). (B) qPCR was used to measure the relative expression levels of Fzd4 in normal and osteogenic induced TDSCs during 3 weeks (*n* = 3/group). (C) StarBase software predicted the targeting sequence of miR-199a-5p. (D) TDSCs were transfected with Fzd4 3’UTR wt or Fzd4 3’UTR mut and co-treated with agomiR-NC or agomiR-199-5p, Relative luciferase was detected using dual luciferase reporter genes (*n* = 3/group). (E-K) TDSCs from C57BL/6J male mice were transfected with agomiR-NC, agomiR-199a-5p, agomiR-199a-5p +vector, agomiR-199a-5p+OV-Fzd4 for 2 days. (E) Relative mRNA levels of Runx2, Opn and β-catenin were analysed by qPCR (*n* = 3/group). (F) Runx2, Opn and β-catenin protein levels analysed by western blots (*n* = 3/group). (F) Immunofluorescence analysis of the expression levels of Runx2, Opn and β-catenin after transfection and 3 days of osteogenic induction, scale bar: 50 μm (*n* = 3/group). (H-K) After transfecting the plasmid and osteogenic induction 7–14 days later, (H) alkaline phosphatase and (G) alizarin red staining was performed. (I) Alkaline phosphatase (*n* = 3/group) and (K) alizarin red (*n* = 3/group) quantification. *p < 0.05; **p < 0.01; ***p < 0.001; ****p < 0.0001.

Compared to sham-operated tendon tissues, a significant elevation of Fzd4 levels in HO tissues by qPCR assay (Figure 3A). Compared to TDSCs cultured in standard growth medium, TDSCs cultured in osteogenic induction medium exhibited a progressively increasing temporal expression pattern of Fzd4 from weeks 0 to 3 (Figure 3B). The mechanism of miRNA action on target genes has been a subject of great interest for many researchers. Lin-4 and let-7, two miRNAs that were identified early on, are thought to hinder the translation of mRNAs by binding partially and complementarily to the 3’UTR of genes’ mRNA [26]. Therefore, to delve deeper into the downstream molecular events following miRNA action, it was predicted that Fzd4 binds to miR-199a-5p by StarBase (Figure 3C). Previous reports indicated that the Fzd4/β-catenin pathway is associated with osteogenesis. To investigate the direct modulation of Fzd4 by miR-199a-5p, a luciferase reporter gene assay was conducted, demonstrating that upregulation of miR-199a-5p decreased luciferase activity induced by 3’ UTR-Fzd4-WT, with no effect on 3’ UTR-Fzd4-MUT luciferase activity (Figure 3D).

In order to explore the impact of miR-199a-5p on the osteogenic differentiation of TDSCs through suppressing Fzd4, by manipulating the expression of miR-199a-5p in osteogenically induced TDSCs using agomiR-199a-5p and increase ing Fzd4 expression with OV-Fzd4, successful overexpression was demonstrated via qPCR. We performed qPCR assay (Figure 3E), western blot analyses (Figure 3F), and immunofluorescence (Figure 3G). The results indicated that treatment with agomiR-199a-5p reduced the expression of Opn, Runx2, and β-catenin, which was countered by OV-Fzd4. The alkaline phosphatase (Figure 3H,I) and alizarin red staining showed resemblance (Figure 3J,K), indicating that miR-199a-5p influences the osteogenic differentiation of TDSCs through the Fzd4/β-catenin pathway.

### The miR-199a-5p-FZD4/β-catenin axis influences the osteogenic differentiation of TDSCs through the regulation of Snhg12 in vivo

To elucidate the impact of the Snhg12-miR-199a-5p-Fzd4/β-catenin pathway on heterotopic ossification in vivo, we administered virus locally and introduced agomiR-199a-5p to modulate the levels of Snhg12, miR-199a-5p, and Fzd4 in mice. H&E staining and micro-CT revealed a significant increase of HO in the group treated with OV-Snhg12 compared to the vector group.

**Figure 4.**
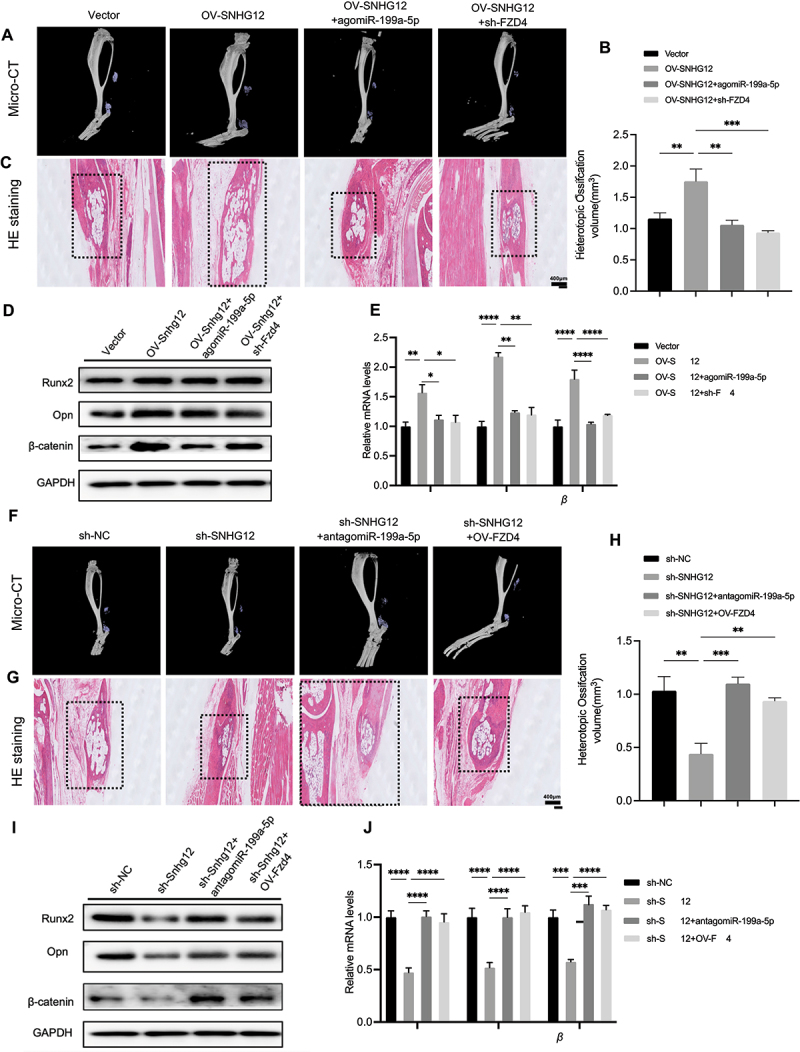
In vivo Snhg12 regulates HO via the miR-199a-5p-Fzd4/β-catenin axis. (A-E) The trauma-induce mice were treated with vector, OV-Snhg12, OV-Snhg12+agomiR-199a-5p, OV-Snhg12+sh-Fzd4 (*n* = 10/group). (A) Micro-CT and (C) H&E staining to detect HO formation in tendon tissues at 10 weeks after transfection, the regions of the tendon: proximal segment (vector), proximal segment (OV-Snhg12), distal segment (AgomiR-199a-5p+OV-Snhg12), and proximal segment (sh-Fzd4+OV-Snhg12), scale bar: 400 μm (three samples from each group). (B) HO volume quantification for each group (three samples from each group). (D) Runx2, Opn and β-catenin protein levels were analysed by western blots (three samples from each group). (E) Relative mRNA levels of Runx2, Opn and β-catenin were analysed by qPCR (three samples from each group). (F-J) The trauma-mice were treated with sh-NC, sh-Snhg12, sh-Snhg12+antagomiR-miR-199a-5p, sh-Snhg12+OV-Fzd4 (*n* = 10/group). (F) Micro-CT and (G) H&E staining to detect HO formation in tendon tissues at 10 weeks after transfection, the regions of the tendon: proximal segment (sh-NC), proximal segment (sh-Snhg12), distal segment (AntagomiR-199a-5p+sh-Snhg12), and proximal segment (OV-Fzd4+sh-Snhg12), scale bar: 400 μm (three samples from each group). (H) HO volume quantification for each group (three samples from each group). (I) Runx2, Opn and β-catenin protein levels were analysed by western blots (three samples from each group). (J) Relative mRNA levels of Runx2, Opn and β-catenin were analysed by qPCR (three samples from each group). *p < 0.05; **p < 0.01; ***p < 0.001; ****p < 0.0001.

**Figure 5.**
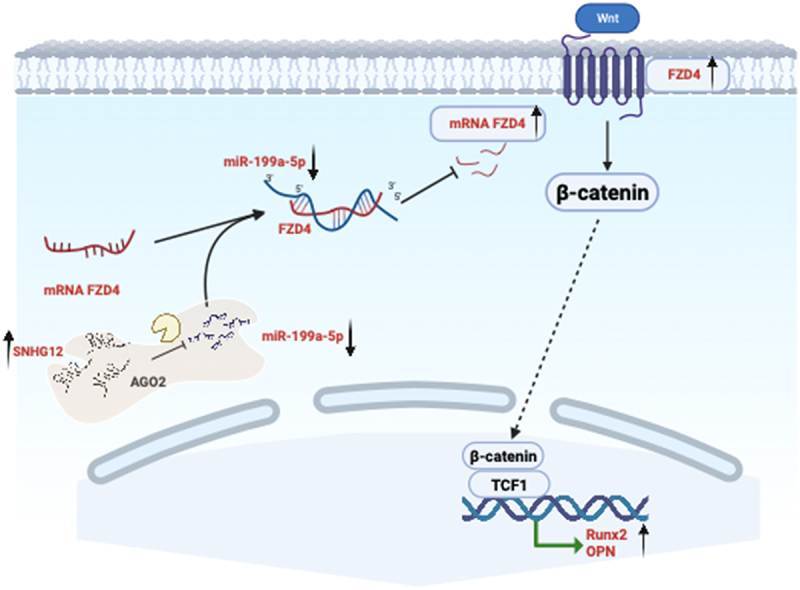
Snhg12 targets miR-199a-5p to regulate osteogenic differentiation of TDSCs via the Fzd4/Wnt/β-catenin pathway.

Nevertheless, by increasing miR-199-5p expression or knockdown Fzd4 led to reduction of HO (Figure 4A–C). Western blots (Figure 4D) and qPCR assays (Figure 4E) showed up-regulation of biological marker Runx2 and Opn expression treated with OV-Snhg12, which but increasing the miR-199-5p expression level or reducing Fzd4 reversed the up-regulation. Subsequent H&E staining and micro-CT of the sh-Snhg12 group showed a significant decrease in HO compared to the sh-NC group, and this decrease could be reverted by altering the expression of miR-199-5p or by upregulating Fzd4 (Figure 4F–H). Western blots (Figure 4I) and qPCR assays (Figure 4J) had similar results. The Snhg12-miR-199a-5p-Fzd4/β-catenin pathway plays a crucial role in HO (Figure 5).

The lncRNA SNHG12, in conjunction with the assistance of Ago protein, binds to miR-199a-5p, thereby preventing the down-regulation of the target gene Fzd4 by miR-199a-5p. This subsequently results in the activation of the Fzd4/Wnt/β-catenin pathway, facilitating the expression of osteogenic-related proteins and augmenting osteogenic differentiation.

## Discussion

HO has been a long-standing subject of investigation, however, a comprehensive understanding of its underlying mechanisms has remained elusive. Effective therapeutic interventions for this clinical challenge are currently lacking, with surgical interventions serving as the primary treatment modality [27]. In particular, HO within tendon structures is often linked to increased tendon stiffness and augmented risk of tendon rupture [28]. TDSCs have been considered to be mainly responsible for injury repair and regeneration of tendon tissue until TDSCs with multi-differentiation potential were isolated from tendon tissue and identified [29]. Furthermore, some scholars have postulated that HO in tendons may result from aberrant differentiation of precursor cells triggered by disturbances in local or systemic factors [30]. Previous in vivo and in vitro studies of TDSCs have revealed that the elevation of bone morphogenetic proteins in TDSCs contributes to their differentiation towards osteogenesis, consequently enhancing healing of the Achilles tendon [31]. In addition, a subpopulation of TDSCs, marked by Ctsk-Cre, has been reported to have a strong osteogenic potential and promote HO in vivo [32]. Based on previous studies, we targeted TDSCs to elucidate their role in HO pathogenesis and the underlying molecular machinery. In this study, through high-throughput sequencing of trauma-induced HO tissues and sham-operated tendon tissues, we identified a significant upregulation of Snhg12 in the HO tissues. Hence, the investigation focused on the impact of Snhg12 on the differentiation of TDSCs into osteogenic cells and its involvement in the development of HO. In vivo, traumatic tendon tissues with Snhg12 overexpression mediated by AAV exhibited upregulation of osteogenic protein markers, Runx2 and Opn. Micro-CT imaging and H&E staining demonstrated promotion of HO formation, whereas silencing Snhg12 yielded contrasting outcomes.

Due to the diverse biological functions of long non-coding RNAs, various hypotheses on their mechanisms of action, for instance, lncRNAs performed biological functions by interacting with proteins and RNAs to form various modules (ceRNA) [33], were key components of cellular address codes, and were intra- and extracellular signalling molecules [34]. Among these, the ceRNA mechanism has been extensively studied and reported. This mechanism involves the regulation of target mRNA by miRNAs, which typically bind to the 3’-untranslated region (3’−UTR) of the mRNA, thereby inhibiting mRNA translation with precision. Thus, the ce-RNA mechanism is the key mechanism for us to study. Consequently, biological software was employed to predict the downstream miRNAs of Snhg12 and the targets of these miRNAs. As a result, several downstream miRNAs of Snhg12, such as miR-129-5p and miR-134-5p, were identified, and miR-199a-5p, along with previous studies indicating its involvement in osteogenic differentiation, was selected for further examination. In previous studies, it was found that the overexpression of miR-199a-5p in human mesenchymal stem cells (MSCs) promoted their osteogenic differentiation [35]. Additional investigation uncovered that Fzd4, situated after miR-199a-5p, has an important function in the Wnt/β-catenin pathway, recognized for its involvement in osteogenic differentiation [36]. A different research demonstrated that LINC01119 has an inhibitory effect on the osteogenic differentiation of MSCs by specifically targeting Fzd4 [37]. The findings suggest that miR-199a-5p plays a role in the osteogenic differentiation by controlling the expression of Fzd4 to modulate the Wnt/β-catenin pathway. Therefore, the aim of this study was to explore the regulatory interplay involving lncRNA Snhg12, miR-199a-5p, and the Fzd4/Wnt/β-catenin pathway in HO. To further confirm the relationship between Snhg12 and miR-199a-5p, at first, Snhg12 was located in the cytoplasm through fish assay, then RIP assay, and luciferase reporter gene assay, which suggested a direct binding interaction between Snhg12 and miR-199a-5p. The findings offer proof of a detrimental governing connection between Snhg12 and miR-199a-5p. Treatment with OV-Snhg12 aggravated osteogenic differentiation of TDSCs, while this effect was reversed with agomiR-199a-5p, suggesting Snhg12 promotes osteogenic differentiation of TDSCs through miR-199a-5p. Additionally, qPCR and western blots revealed an increase in Fzd4 expression following the suppression of miR-199a-5p. Earlier studies have also indicated that Fzd4 is involved in the regulation of osteogenic differentiation via the Wnt/β-catenin pathway. In TDSC, treatment with agomiR-199a-5p exacerbated the osteogenic differentiation of TDSCs, the effect that was counteracted by overexpressing Fzd4. The experimental treatments on TDSCs yielded similar results in mice. Moreover, functional recovery experiments in mice revealed that knockdown of Snhg12 induced by AAV inhibited HO formation, the effect that was negated by antagomiR-199a-5p treatment or overexpressing Fzd4.

## Conclusion

This study validated Snhg12 targets miR-199a-5p to regulate osteogenic differentiation of TDSCs via the Fzd4/Wnt/β-catenin Pathway.

## Abbreviations

HO: Heterotopic ossification
TDSCs: Tendon stem cells
BMSCs: Bone marrow mesenchymal cells
Snhg12: small nucleolar RNA host gene 12
Fzd4: Frizzled class receptor 4
qRT-PCR: Quantitative reserve-transcriptase polymerase chain reaction
FBS: Foetal Bovine Serum
AAV9: Adeno-associated virus 9
EDTA: Ethylenediaminetetraacetic acid
FISH: Fluorescence in situ hybridization
micro-CT: Micro-computed tomography
BCA: Bicinchoninic acid
TBST: Tris buffered saline tween
RIP: RNA Immunoprecipitation
H&E: Haematoxylin and eosin
PBS: Phosphate-bufered saline

## Limitations

First, the specificity of the inhibitors and activators used in this study needs to be further investigated, and they cannot completely mimic the normal physiological state; the specific mechanism of action in vivo is not clear when the inhibitors and activators are injected directly into the mice in situ, and the pharmacokinetics and drug dispersion cannot be detected. Secondly, the direction of clinical translation of this study is not clear, and the clinical use of miRNA and lncRAN is relatively immature.

## Supplementary Material

Supplementary_materals.docx

## Data Availability

RNA-seq data that support the findings of this study are available in [Gene Expression Omnibus database at https://www.ncbi.nlm.nih.gov/geo/query/acc.cgi?acc=GSE233201], reference number. Other data that support the findings of this study are available from the corresponding author upon reasonable request.
