# Supplementary material for: Snhg12 targets miR-199a-5p to regulate osteogenic differentiation of TDSCs via the Fzd4/Wnt/β-catenin pathway
Source: RNA Biol. 2025 Jun 24;22(1):1–13. doi: 10.1080/15476286.2025.2518754 (PMC12710899; doi:10.1080/15476286.2025.2518754)
Supplement: Supplementary_materals.docx [file KRNB_A_2518754_SM5383.docx]

**Table. S1 The sequences of shRNA.**

| Virus | sequences |
| --- | --- |
| AAV-GFP | GAAGTCGTGAGAAGTAGAA |
| AAV-shFzd4 (ID:14366) | GGAAGGACCAGGTGATGAAGA |
| AAV-shSnhg12 (ID: 100039864) | ACCAGTGAAGCAGCCATTATA |

**Table. S2 The primer sequences for RT-qPCR**

| Gene | Forward sequence | Reverse sequence | ID |
| --- | --- | --- | --- |
| Snhg12 | GGTGCTCCAGGCAATAACT | CTCCCATACAGTCCGAACAT | 100039864 |
| miR-199a-5p | CCCAGUGUUCAGACUACCUGUUC | GAACAGGAGUCUGAACACUGGG | 387194 |
| Fzd4 | ACTTTCACGCCGCTCATCCAGT | TGCGACACTTGGTCAGGACTCT | 14366 |
| Runx2 | CCTGAACTCTGCACCAAGTCCT | TCATCTGGCTCAGATAGGAGGG | 12393 |
| Opn | GCTTGGCTTATGGACTGAGGTC | CCTTAGACTCACCGCTCTTCATG | 20750 |
| Actb | CATTGCTGACAGGATGCAGAAGG | TGCTGGAAGGTGGACAGTGAGG | 11461 |
